# Using Nanoimprint Lithography to Create Robust, Buoyant, Superhydrophobic PVB/SiO_2_ Coatings on wood Surfaces Inspired by Red roses petal

**DOI:** 10.1038/s41598-019-46337-y

**Published:** 2019-07-10

**Authors:** Yushan Yang, Haishan He, Yougui Li, Jian Qiu

**Affiliations:** 1 College of Materials Science and Engineering, Southwest Forestry University, Yunnan Kunming, 650224 People’s Republic of China; 2 Wood Collection, Southwest Forestry University, Yunnan Kunming, 650224 People’s Republic of China

**Keywords:** Nanoscale materials, Biosurfaces, Nanoscale materials, Biosurfaces, Supramolecular polymers

## Abstract

Robust, buoyant, superhydrophobic PVB/SiO_2_ coatings were successfully formed on wood surface through a one-step solvothermal method and a nanoimprint lithography method. The as-prepared PVB/SiO_2_/wood specimens were characterized by scanning electron microscopy (SEM), X-ray diffraction (XRD), Fourier transform infrared (FT-IR), thermogravimetric/differential thermogravimetric (TG–DTG) analyses. The superhydrophobic property and abrasion resistance of rose-petal-like wood were measured and assessed by water contact angle (WCA) and sand abrasion tests. The results show that PVB/SiO_2_/wood not only exhibited a robust superhydrophobic performance with a WCA of 160° but also had excellent durability and thermostability during the sand abrasion tests and against corrosive liquids. Additionally, the as-prepared PVB/SiO_2_/wood specimens show high buoyancy.

## Introduction

Natural wood, a low-cost and earth-abundant material, is ubiquitously used as a structural material across the globe^1,2^. Historically, wood timber has been extensively applied in vzrious daily applications, such as construction, indoor decoration, translation mining, transportation, furniture^3^, etc. however, it is susceptible to water absorption or water vapor, resulting in cracking, deformation, decay and insect-damage^4,5^, and thus, the application area is limited. One possible efficient solution would be to prepare a superhydrophobic coating for the surfaces of wood timber to prevent water adsorption^3,6^. Over the past few years, some interesting coatings with various properties, such as self-cleaning, anti-corrosion, superhydrophobic or fire resistance properties, have been studied^5–7^. However, most researchers reported only the method of superhydrophobic wood analysis, and only a few robust superhydrophobic wood surfaces have been developed^6^. However, few researchers have focused on superhydrophobic wood surface-outgrowth-induced high buoyancy performance. Nevertheless, developing robust, buoyant, superhydrophobic surfaces on wood substrates with a superhydrophobic coating will have great potential advantage for theoretical research and practical applications.

Humans can learn from nature. After billions of years of evolution, nature creates countless mysterious living organosms that demonstrate almost perfect structures and functions. Learning from nature, many researchers have discovered many functional interfacial materials for applications, including materials with zwitter-wettability, superhydrophobicity, adhesion, self-cleaning, anti-snow, anti-fogging/icing, anti-oxidation, and corrosion-resistancepropeerties^8–18^. The example in nature include the self-cleaning superhydrophobic surface of the lotus leaf, toro leaf, and water lily^3,18–20^; the directional catchment effect in a spider web^21^; directional adhesive and self-cleaning superhydrophobic cicada’ wings, butterfly wings, and peacock feathers^22,23^ anisotropic superhydrophobic rice leaves^24,25^; high adhesion superhydrophobic peanut leaves^26,27^; the superhydrophobic, high adhesive, and reversibly adhesive gecko foot^26^; anti-freezing penguin’ wings^28^; the robust, superhydrophobic water strider leg^29,30^; the superhydrophobic, highly adhesive, and structural colour red rose petals^31^. Inspired by the approaches in nature, we will develop strategies to design and fabricate micro-nanoscale wood surfaces with superwettability to prevent the loss of performance in outdoor environments. Recently, more attention has been paid to the fabrication of superhydrophobic wood surface inspired by biological materials^7^. A variety of methods, such as hydrothermal, solvothermal, soft-lithography, sol−gel, photolithography, spraying, template, layer-by-layer, and self-assembly, have been used to replicate the biomimetic micro/nanostructures of surfaces^3,7,8,32–34^. As a technique for preparing micro/nanostructures, a solvothermal treatment combined with nanoimprint lithography methods can overcome many of the shortcomings of the above methods. To the best of our knowledge, there are no reports about the fabrication of a superhydrophobic surface with outgrowth-induced high buoyancy on wood by a two-step method of solvothermal deposition and nanoimprinted lithography.

In this study, a petal-like PVB/SiO_2_ superhydrophobic wood was successfully fabricated by using red rose petals, and cross-linked PDMS was used as the master template and stamp for nanoimprinted lithography after solvothermal deposition. The as-prepared PVB/SiO_2_/wood surface not only had robust superhydrophobic performance during the ultrasonic rinse and sand abrasion tests, but also stable super-repellency towards commonly used liquids, including brine, tea, milk and vinegar. Meanwhile, the buoyancy of a superhydrophobic surface is a very important influence on the properties of the material. Therefore, petal-like PVB/SiO_2_ superhydrophobic wood could effectively prevent moisture from penetrating wood and improve the dimensional stability, which can satisfy our daily life application needs

## Results and Discussion

The mechanism of the condensation reaction of hydrophobic monodispersed nano-SiO_2_ microspheres can be expressed by the reaction in Equations (1)–(3):

1 $${\rm{Si}}\mbox{--}{({{\rm{OC}}}_{2}{{\rm{H}}}_{5})}_{4}+4{{\rm{H}}}_{2}{\rm{O}}+{{\rm{NH}}}_{3}\cdot {{\rm{H}}}_{2}{\rm{O}}\to {\rm{Si}}\mbox{--}{({\rm{OH}})}_{4}+4\,{{\rm{C}}}_{2}{{\rm{H}}}_{5}{\rm{OH}}$$

2 $${\rm{Si}}\mbox{--}{({\rm{OH}})}_{4}+{\rm{Si}}\mbox{--}{({{\rm{OC}}}_{2}{{\rm{H}}}_{5})}_{4}\to \equiv {\rm{Si}}\mbox{--}{\rm{O}}\mbox{--}{\rm{Si}}\equiv +4\,{{\rm{C}}}_{2}{{\rm{H}}}_{5}{\rm{OH}}$$

3 $${\rm{Si}}\mbox{--}{({\rm{OH}})}_{4}+{\rm{Si}}\mbox{--}{({\rm{OH}})}_{4}\to {\rm{Si}}\mbox{--}{\rm{O}}\mbox{--}{\rm{Si}}\equiv +4{{\rm{H}}}_{2}{\rm{O}}$$

**Figure 1:**
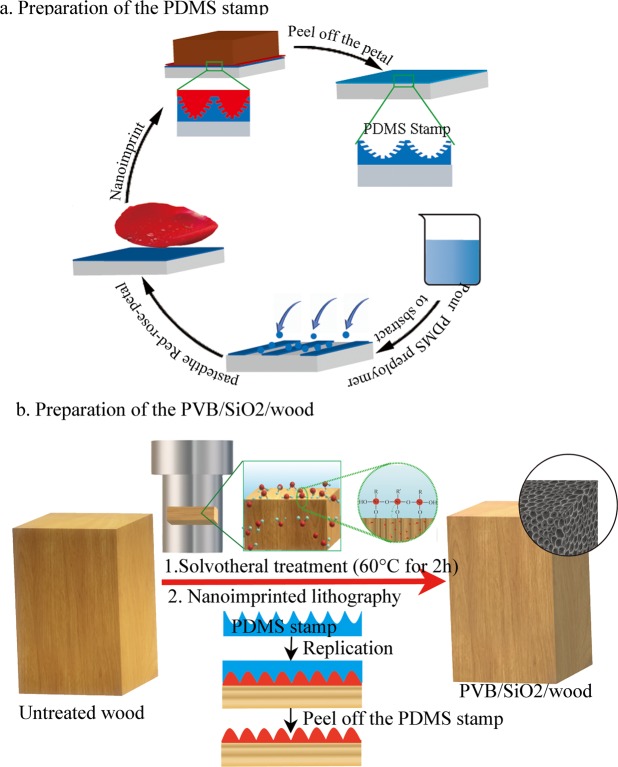
Schematic illustration of (**a**) the replication process of the PDMS stamp with a negative nanostructures of biomimetic red rose petals, (**b**) the fabrication process for PVB/SiO_2_/wood.

According to previous results, a possible mechanism could be as follows. First, ethyl orthosilicate is hydrolysed to SiO_2_ in aqueous ammonia. Hydrophobic monodisperse nano-SiO_2_ microspheres are formed by the alcohol condensation and water condensation reaction. On the basis of the results above, a schematic illustration of the process of a replicating a biomimetic red rose petal through nanoimprint lithography is depicted in Fig. 1a. First, a mixture of curing agent and PDMS prepolymer (Sylgard 184 Silicone Elastomer Kit, Dow Corning) with a weight ratio of 1:10 was cast onto a fresh red rose petal to prepare the PDMS stamp, and the thickness was 3 mm. Then, the stamp was degassed in a vacuum container to remove air bubbles under the petal. After curing at 70 °C for 4 h, the solidified PDMS stamp was peeled off from the master template; thus, inverse petal structures were obtained. Tthen, (Fig. 1b) natural wood was placed into the precursor PVB/SiO_2_ solutions at 60 °C for 24 h in a Teflon-lined autoclave to increase bonding strength via furthersolvothermal treatment. This coating was plated on a natural wood surface, with the same replicate process, but the inverse petal structure cross-linked PDMS stamp was used as the master template to produce a petal-like structure. Finally, the PDMS template was peeled off the wood surface, and biomimetic PVB/SiO_2_/wood was fabricated.

**Figure 2:**
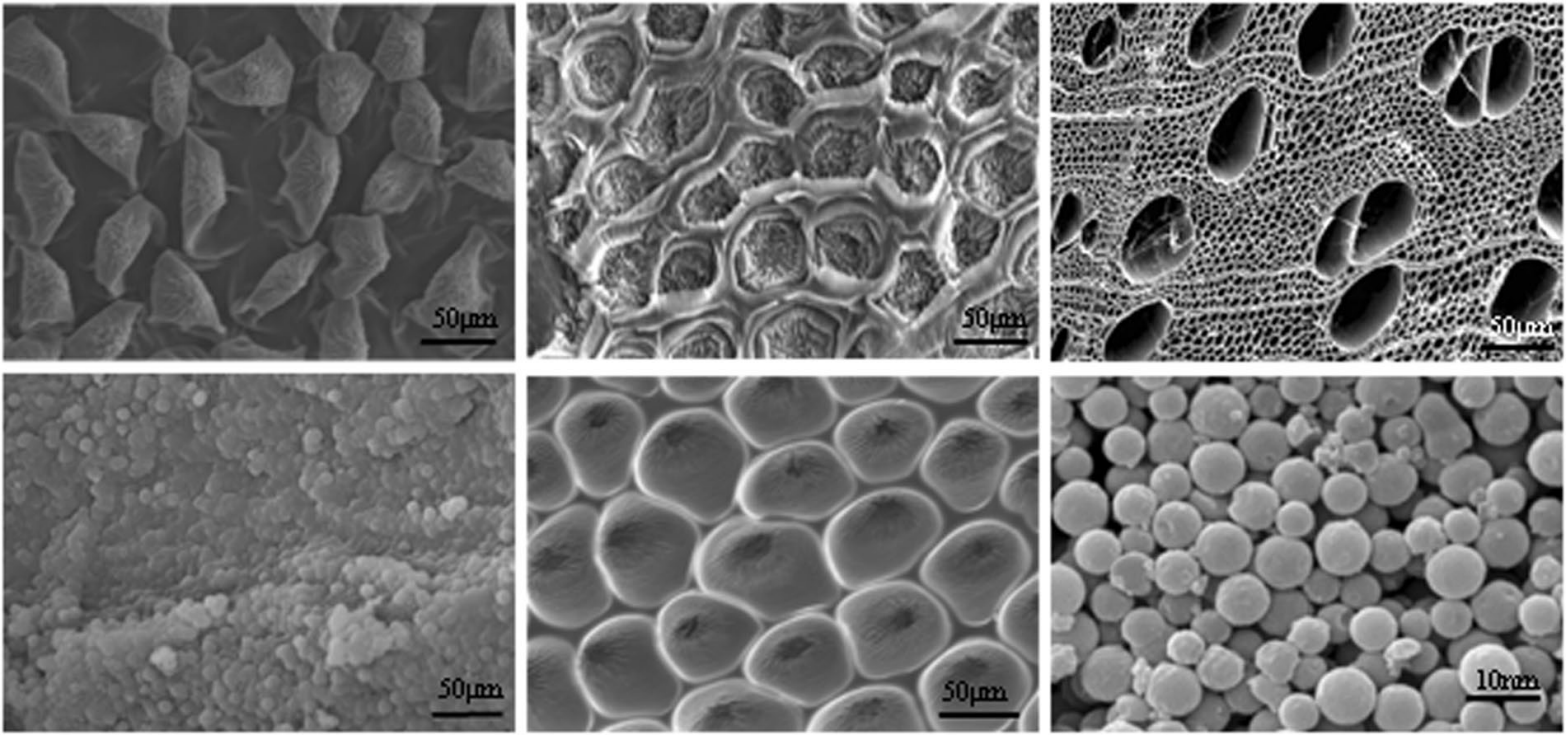
SEM images of the surface of red rose petals surface (**a**), inverse petal structures PDMS stamp (**b**,**c**) natural wood, PVB/SiO_2_ coating (**d**), and biomimetic PVB/SiO_2_/wood (**e**,**f**) with different magnifcation.

Figure 2a shows SEM images of the red rose petal, inverse petal structure PDMS stamp, untreated wood, and biomimetic PVB/SiO_2_/wood. In Fig. 2a, the pristine red rose petal surface is covered by micro/nano-papillae with grooves and folds at the top of each papilla. This micro/nanostructure on the red rose petal surface allows for high adhesive superhydrophobicity because of the large adhesive force between the liquid droplet and the red roses petal surface. Figure 2b shows the SEM images of the as-prepared PDMS stamp surface, and the inverse petal micro/nanobiomimetic structures were observed on the surface of the red rose petal. In Fig. 2c, many traditional open vessel-to-vessel mesoporous structures can be observed on natural wood surface in a across section. After solvothermal treatment, the surface of the untreated wood was uniformly covered by a compact PVB/SiO_2_ coating, as shown in Fig. 2d. After the second nanoimprint lithography step, Fig. 2e shows that micro/nano-papillae and nanofolds were found on the PVB/SiO_2_/wood surface. The top of each micro/nano-papilla includes nanofolds oriented towards the centre. The surface topography of the rose-petal-like PVB/SiO_2_/wood was similar to that of the fresh red rose petal surface. In addition, the small, superhydrophobic, monodispersed nano-SiO_2_ microspheres can be found in the higher-magnification images (Fig. 2f). Therefore, these results indicate that the nanoimprint lithography method successfully reproduced and retained the macrostructure and micro-nanostructure of red roses petal onto the wood surface.

**Figure 3:**
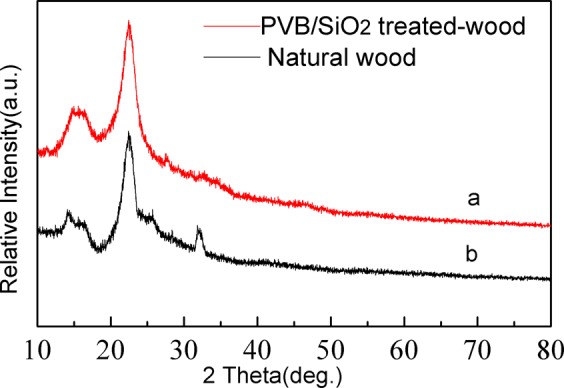
XRD patterns of (**a**) natural wood and (**b**) PVB/SiO_2_/wood.

Figure 3 displays the XRD patterns of the untreated wood and PVB/SiO_2_/wood. In Fig. 3a, two strong characteristic crystalline diffraction peaks located at approximately 15° and 22° appear in the natural wood spectrum, and originate from the crystalline region of cellulose in natural wood. There was no other obvious characteristic peaks^2,35^. As shown in Fig. 3b, new strong diffraction peaks were observed after treatment, and these diffraction peaks at 34° could be well-indexed to the standard diffraction pattern of nanostructure SiO_2_ (JCPDS 46-1045), except for the characteristic peaks of the untreated wood. This result suggests that the as-prepared PVB/SiO_2_ solution had a high purity and no impurities.

**Figure 4:**
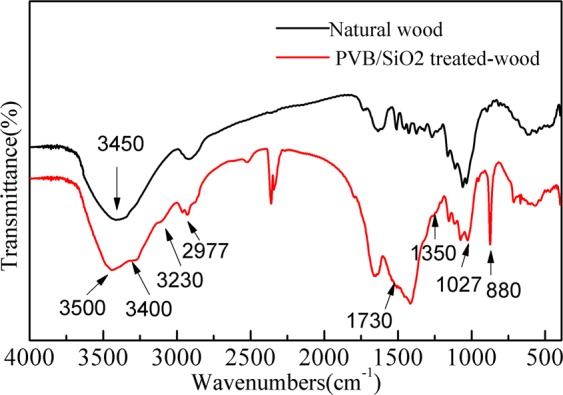
FTIR spectra of (**a**) natural wood and (**b**) the PVB/SiO_2_/wood.

Figure 4 shows the FTIR spectra of the untreated wood and PVB/SiO_2_/wood. As shown in Fig. 4a, the main absorption bands in the FTIR spectra are located at ~3450 cm^−1^, ~2908 cm^−1^, ~1700 cm^−1^ and ~1429 cm^−1^, corresponding to the O–H, C–O, C=O and C–H_3_ stretching vibrations, respectively, and are attributed to the untreated wood. In Fig. 4b, the main absorption peaks of PVB/SiO_2_/wood at 3500 cm^−1^ are attributed to the stretching vibration of the hydroxyl groups (-OH). The absorption peaks at 3400 cm^−1^ become increasingly stronger and are mainly attributed to the stretching vibration of silicon hydroxyl groups. This result indicates that more -OH reacted with the superhydrophobic PVB/SiO_2_ coating. The C–H_3_ stretching vibration absorption at 2977 cm^−1^ and stronger absorption can be due to methyl groups. The absorption peaks at 1350 cm^−1^ indicate the stretching vibrations of C-F groups, and the peak at 1730 cm^−1^ corresponds to the C=O bond stretching vibrations. In addition, the strong absorption peaks at 810 cm^−1^ can be assigned to Si-O-Si and Si–CH_3_, which indicated that the monodispersed SiO_2_ microspheres were deposited onto the wood surface and enhanced the untreated wood hydrophobic performance. The results demonstrate that the PVB/SiO_2_ coating was successfully placed on the wood surface and existence of a long-chain-alkyl group on the surface, both of which were obtained through a one-step solvothermal method and a nanoimprint lithography method.

**Figure 5:**
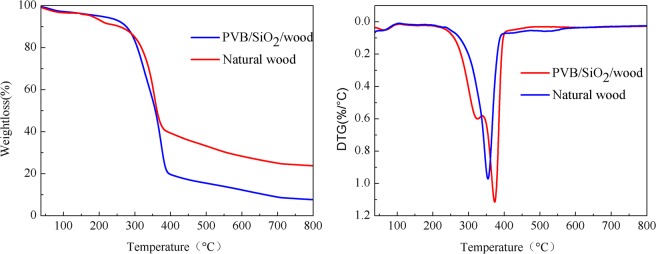
TG-DTA curves of (**a**) natural wood and (**b**) the PVB/SiO_2_/wood.

The thermogravimetric and differential thermogravimetric analysis (TG–DTG) curves of the untreated wood and PVB/SiO_2_/wood are shown in Fig. 5. As shown in Fig. 5a, a small weight loss of 2–3% was observed at 37–170 °C in both samples, which was attributed to the evaporation of adsorbed water. After the combined PVB/SiO_2_ coating was added onto the wood surface, the initial decomposition temperature of PVB/SiO_2_/wood was approximately 303 °C, which was 30.5 °C higher than that of the untreated wood^35,36^. In Fig. 5b, the maximum pyrolysis rate of PVB/SiO_2_/wood occurred at 345 °C, and the pyrolysis rate was 51% lower than that the untreated wood, which might be attributed to depositing the PVB/SiO_2_ coating on the wood surface. During the whole process of weight loss, the mass percentage of pyrolysis residue for untreated wood and PVB/SiO_2_/wood was approximately 8.9% and 29.8%, respectively. These results show that the thermal stability of PVB/SiO_2_/wood improved because of the strong interaction between the natural wood and the PVB/SiO_2_ coating.

**Figure 6:**
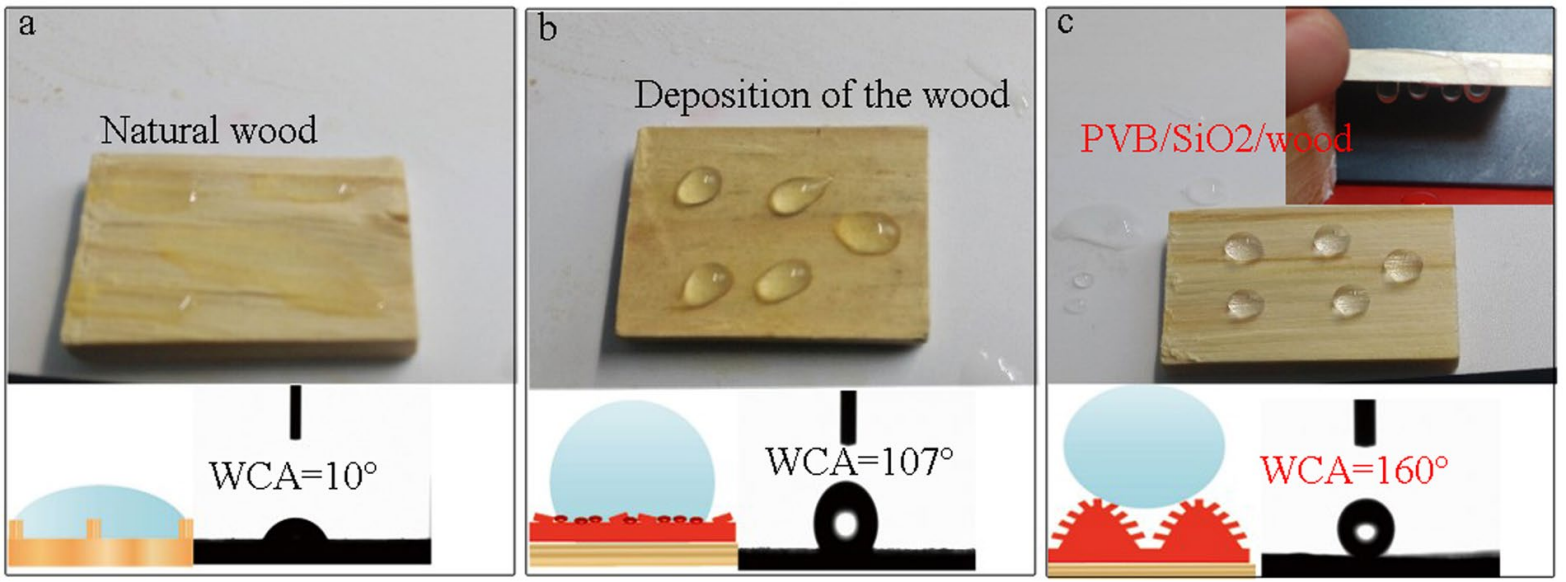
Superwettability of (**a**) natural wood, (**b**) wood with PVB/SiO_2_ coating, and (**c**) PVB/SiO_2_/wood.

The surface wettability of the as-prepared PVB/SiO_2_/wood specimens were evaluated by WCA measurements with a volume of approximately 5 μL for the water droplets. Figure 6a shows the water droplets behavior on the untreated wood surface. The surface had a hydrophilic performances with a WCA of 10°. Such a small WCA was attributed to the hydroxyl groups on the natural wood surface and the low surface energy of open lumina of wood. Without nanoimprint lithography(Fig. 6b), the solvothermal deposition of PVB/SiO_2_ coating exhibited hydrophobic properties with a WCA of 107°. When a droplet was dropped onto the as-prepared PVB/SiO_2_/wood specimens surface was formed an approximate sphere, and couldn’t roll-off even when the wood was turned upside down (the inset). And the WCA value measured on the surface of PVB/SiO_2_/Wood was 160° (Fig. 6c). The results show that the superhydrophilic surface of the natural wood was directly transformed into a superhydrophobic surface after solvothermal treatment with nanoimprint lithography. The Cassie and Baxter equation can theoretically be used to explain the superhydrophobicity of the as-prepared PVB/SiO_2_/wood.

**Figure 7:**
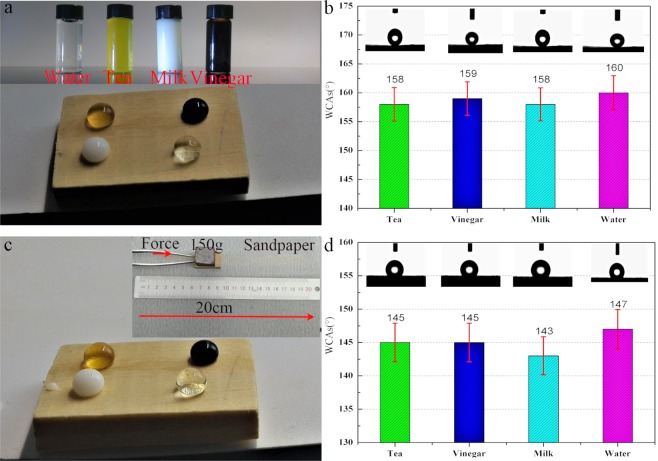
Robust superamphiphobic performances of PVB/SiO_2_/wood measured by (**a**) an ultrasonic rinse for 24 h and (**b**) a sand paper abrasion test. (**c**,**d**) The corresponding WCAs of the PVB/SiO_2_/wood with liquid droplets of brine, tea, milk and vinegar.

To confirm the stability and robust superhydrophobic properties of PVB/SiO_2_/wood, 4 types of liquids, including brine, tea, milk, and vinegar, were used to examine the surface repellency toward commonly used liquids after an ultrasonically rinsed test for 24 h (Fig. 7a). In addition, sandpaper abrasion tests were performed using awood surface weighting 150 g was, which was placed on sandpaper (standard sandpaper, grit no. 320 cW) and moved 20 cm along a ruler (Fig. 7c). As shown in Fig. 7a,c, it is obvious that the surface of PVB/SiO_2_/wood repels the tested liquids, that is, the WCAs were closed to 150° under harsh conditions after various artificially accelerated ageing tests (Fig. 7b,d). The high WCAs of the PVB/SiO_2_/wood surface can be attributed to the PVB/SiO_2_ coating, which significantly demonstrated a robust superhydrophobic performance.

**Figure 8:**
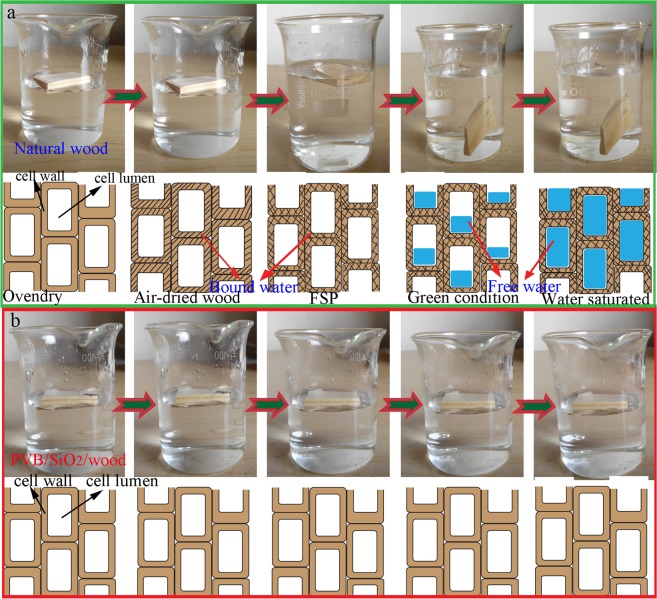
Water absorption properties of (**a**) natural wood and (**b**) PVB/SiO_2_/wood, which are consistent with the schematic illustration of water absorption over 45 days.

Water absorption, a crucial characteristic of natural wood, determines the ultimate applications. In this study, the water resistance of natural wood and PVB/SiO_2_/wood was investigated. The experiments were carried out by immersing natural wood and PVB/SiO2/wood samples in water for 45 days. As shown in Fig. 8a, the natural wood sample sank within 45 days after the specimen was fully immersed in water. The water absorption processes appeared during the typical five condition over the range of floating on the water surface to sinking into water. However, the PVB/SiO_2_/wood samples floated on the water surface for 45 days (Fig. 8b). These performances can be attributed to the superhydrophobic paint coating, including the wood density and moisture content of the wood. The density of the cell wall material is approximately 1.5 g/cm^3^, whereas wood has a density of less than 1.0 g/cm^3^, allowing floating in water. Moisture can exist in natural wood as free water in cell lumens and as bound water within cell walls^37^. When the moisture content in both is saturated with water, natural wood samples will sink in water.

**Figure 9:**
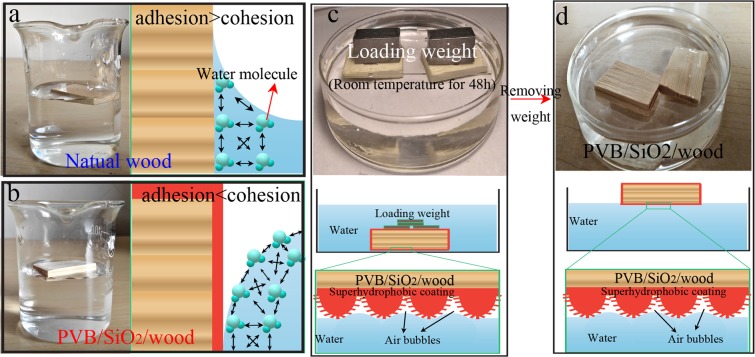
Schematic illustration of the adhesion and cohesion forces of water molecules on (**a**) natural wood and (**b**) PVB/SiO_2_/wood surface. Supporting buoyant forces of PVB/SiO_2_/wood (water surface tension and air bubbles) applied to whole surface-coated samples after loading with weight. (**c**,**d**) Schematic illustration of the PVB/SiO_2_/wood sample in water after loading and removal loading weight.

To determine the buoyancy of PVB/SiO_2_/wood induced by the superhydrophobic coating, two samples were used. As shown in Fig. 9, the natural wood and PVB/SiO_2_/wood samples that retain wettability had buoyancy. Figure 9a shows that the surface energy of natural wood is higher than that of water, indicating that natural wood tends to attract water. However, PVB/SiO_2_/wood has a lower surface energy than the water. This result indicates that the water cohesion force of PVB/SiO_2_/wood was reduced by the superhydrophobic coating. The water surface close to the PVB/SiO_2_/wood sample has a convex meniscus, showing that the cohesion forces between PVB/SiO_2_/wood and water molecules are greater than the adhesion forces between the water molecules. The PVB/SiO_2_/wood loading ability determined by weight loading tests is showed in Fig. 9c,d. When the load was removed, wood floating was clearly observed on the water surface(Fig. 9d), which was attributed to the water surface tension and number of air bubbles entrapped at the PVB/SiO_2_/wood surface. According to the Cassie-Baxter equation, PVB/SiO_2_/wood can be in an impregnating wetting state which water penetrate the micro/nano-papillae, and the air remaining in the nanofolds. However, the high buoyancy of PVB/SiO_2_/wood could be attributed to the surface of the superhydrophobic PVB/SiO_2_ coating and the embedded air bubbles on the wood samples. Detailed analysis of the surface tension of the air-water interfaces and superhydrophobic coating of the PVB/SiO_2_/wood surface shows excellent buoyancy.

## Conclusion

In conclusion, natural wood with a robust, water resistant, mechanically stable, highly thermostable and highly buoyant performance was successfully fabricated by solvothermal deposition of hydrophobic monodispersed nano-SiO_2_ microspheres, followed by a nanoimprint lithography treatment. This method introduced SiO_2_ microspheres into a PVB solution and plated them onto a wood surface to be replicated. Thus, the resultant not only exhibited superhydrophobic performance with a water contact angle of 160° but also had high buoyancy. In addition, PVB/SiO_2_/wood exhibited excellent superhydrophobic properties in the liquids, sandpaper and ultrasonic tests. PVB/SiO_2_/Wood also presented excellent mechanical stability and thermostable properties.

## Materials and Methods

### Materials

Red roses were picked from Kunming City (Yunnan Province, China). Wood samples (Populus ussuriensis Kom) were cut with a size of 30 mm × 10 mm × 5 mm. Then, the samples were ultrasonically rinsed in acetone several times, then ultrasonically rinsed in deionized water for 15 min and vacuum dried at 105 °C for 24 h. The dry density of the wood was 0.39 ± 0.05 g/cm^3^.

Polyvinyl butyral (PVB, Mw 40,000–70,000), polydimethylsiloxane (PDMS) and curing agent(Sylgard 184 Silicone Elastomer Kit, Dow Corning), acetone, octadecyltrichlorosilane (OTS), absolute alcohol, and ethyl silicate(TEOS) were all purchased from Aladdin (Shanghai, China). All chemicals werev used as received.

### Fabrication of the PVB/SiO_2_ hydrophobic solution

First, 5 g PVB solid was dissolved in 45 mL absolute alcohol at ambient temperature for 0.5 h under magnetic stirring for 30 min, and then, the solution was dissolved at 60 °C for 2 h under magnetic stirring. Then, 1 mL OTS and 2 ml TEOS solution were added into 18.6 mL absolute alcohol solution at room temperature many times under magnetic stirring. Then, 0.5 mL OTS solution and as-prepared PVB solution were added to prepare the composite solution, which was placed in an electrically heated thermostatic oil bath at 60 °C with vigorous magnetic stirring. Then, 0.5 ml of OTS was added, and the PVB/SiO_2_ solution was prepared and solidified at ambient temperature for 24 h prior to further use.

Figure 1a shows a schematic illustration of the replication process of the PDMS stamp with negative nanostructures of biomimetic red rose petals. First, fresh red rose petals were used as a master template and rinsed under deionized water for 1~2 min. Then, the curing agent and PDMS prepolymer with a 1:10 weight ratio were cast onto a fresh red rose petal to fabricate the PDMS stamp by a condensation reaction, and the thickness was approximately 3 mm. Next, the sample was degassed for 1 h, cured by a condensation reaction at 60 °C for 6 h and peeled off the master template of the red rose petal. Finally, the complementary surface topographic structure of the red rose petal was replicated on the cross linked PDMS substrate.

### Fabrication process for a PVB/SiO_2_/wood

Is illustrated in Fig. 1b. Natural wood was placed into the precursor PVB solutions at 60 °C for 24 h in a Teflon-lined autoclave for a continus hydrothermal process (the thickness of the PVB-SiO_2_ coating was approximately 3~5 mm).After that, the micro/nanostructure-array pattern was nanoimprinted on the PVB/SiO_2_ deposited wood surface by PDMS stamp for replication at 60 °C for 4 h in the vacuum dring. Finally, PDMS stamp were peeled off, and PVB/SiO_2_/wood was obtained.

### Characterization

Morphologies of the red rose petals, PDMS stamp and PVB/SiO_2_/wood were observed by scanning electron microscopy (SEM, FEI, Quanta 200.USA) at an accelerating voltage of 12.5 Kw. The crystalline structures of the natural wood and PVB/SiO_2_/wood were identified by X-ray diffraction (XRD, Rigaku D/MAX 2200, Japan), operating with Cu Kα radiation (λ = 1.5418 Å) at 40 kV, 40 mA, in the rang from 10° to 80° (scan rate of 4°/min). The FTIR spectra were recorded for functional groups on a Fourier transform infrared instrument (FT-IR, Magna-IR 560 ESP, Nicolet) in the range of 400–4000 cm^−1^ with a resolution of 4 cm^−1^. The thermal stability performance of the natural wood and PVB/SiO_2_/wood were examined by thermogravimetric and differential thermogravimetric analyses (TG–DTG, SDT Q600, USA); 3.415 mg of the sample was measured at a heating rate of 10 °C/min, a gas flow rate of 50 ml/min in the air with nitrogen, and a temperature range from ambient room temperature to 800 °C. Optical water contact angles of water and other liquids (milk, tea, and vinegar) were measured using an OCA 20 instrument (Data physics, Germany) at ambient temperature. The volume of all liquids was 5 μL. The average WCA value was measured at five different positions of each sample surface.
